# Regulatory and policy implications of sand mining along shallow waters of Njelele River in South Africa

**DOI:** 10.4102/jamba.v11i3.727

**Published:** 2019-07-04

**Authors:** Tendayi Gondo, Humphrey Mathada, Francis Amponsah-Dacosta

**Affiliations:** 1 Department of Urban and Regional Planning, School of Environmental Sciences, University of Venda, Thohoyandou, South Africa; 2 Department of Geography and Geo-Information Sciences, School of Environmental Sciences, University of Venda, Thohoyandou, South Africa; 3 Department of Mining and Environmental Geology, School of Environmental Sciences, University of Venda, Thohoyandou, South Africa

**Keywords:** Sand Mining, Extraction, Environmental Concern, Guidelines, Morphological

## Abstract

The ever-increasing interest in mining of sand in shallow waters of many rural rivers on the one hand and the growing concern for the environment on the other underscore the need to develop better management policies that govern sand extraction. Although literature pointing to increased environmental consciousness by some mining operations exists, the link between environmental concerns and sand mining has however remained a controversial matter and an under-researched area in South Africa. Consequently, decisions relating to what actions should or should not be taken to limit environmental concerns associated with sand mining operations in South Africa are not known. This analysis sought to explore regulatory and policy implications of sand mining operations along a sample of sites of Njelele River in South Africa. Data were gathered through observation, household questionnaire survey and a series of Participatory Rural Appraisal (PRA) exercises conducted with selected community members and sand miners. We used a combination of *K*-means clustering and Discriminant Function Analysis (DFA) to determine the major environmental attributes explaining the state of affairs in sand mining. Regulatory and policy implications were developed using a combination of Gap analysis; Strengths, Weaknesses, Opportunities and Threats (SWOT) analysis; and the development of a Threats, Opportunities, Weaknesses and Strengths (TOWS) matrix strategy. Our analysis identified a series of morphological, ecological, socio-ecological, governance and physical factors that were major areas of concern in three distinct clusters of sand mining sites. We concluded by discussing a number of regulatory and policy implications of sand mining at three scales, namely strategic, institutional and operational scales.

## Introduction

In South Africa, the ever-increasing interest in sand mining and dumping of sand in shallow waters of many rural rivers on the one hand and the growing concern for the environment on the other underscore the need for better understanding of management policies that govern the extraction and dumping of sand. Such concerns are also consistent with a global concern for managing environmental consequences associated with mining industries in general (Hassan & Ibrahim 2012; Warhurst 2001). Although literature pointing to increased environmental consciousness by some mining companies exists, the link between sustainable development and mining has however remained a controversial issue and largely an under-researched area when it comes to identifying resource management scenarios that are likely to breed beneficial environmental outcomes in the small-scale mining sector (Lambert 2001; Warhurst 2001). The main area of concern has been how to regulate and provide guidelines and procedures to mitigate the potential environmental damage from these activities (Trop 2017; UNEP 2014; Uscinowicz et al. 2014).

There is a dearth of conceptually sound analytical studies for estimating environmental damage resulting from sand extraction and associated implications for the development of appropriate management policies (Drucker, Waskes & Byrnes 2004; UNEP 2014). The end result is information with which sound management decisions can be taken is limited. Two greatest challenges as observed by Worrall et al. (2009) and Haberl, Wackernagel and Wrbka (2004) are as follows:

1. How to balance socio-economic benefits and socio-environmental conditions (Worrall et al. 2009)?
2. How to provide useful information to support decision-makers in determining which actions should or should not be taken to effectively improve the sustainability of the mining sector?

This analysis argues that developing effective guidelines to govern sand extraction is critical to fostering environmentally responsible practices among the sand extractors (Vintró, Sanmiquel & Freijo 2014). Such environmentally responsible practices may help minimise negative impacts on the environment. The major aim of this analysis was to identify regulatory areas of concern associated with sand mining that can be used as important precepts in developing an effective regulatory and policy framework for the industry.

### What is sand mining?

Sand mining can be defined as activities associated with extracting sand from the ground. Although sand can be artificially manufactured by crushing coarser aggregates such as stone and gravel mined from a quarry, this analysis focuses on the mining of natural sand from the environment, particularly sand that is found along shallow waters of rivers. Following the cue from Green (2012), we reckon that a sand miner would require basic equipment such as a dozer to clear vegetation and build access roads, an excavator or front-end loader to scoop up sand from the deposit, and trucks to carry the sand away. Such an activity can be set up with relatively low cost, and we envisage that barriers to entry are low.

### Sand mining risks and associated impacts

Most sand mining impacts are associated with the removal of substratum material, alteration of the bottom topography, sediment composition, changes in depth and current strength, and the modification of hydrologic conditions (Allsopp et al. 2013; Uscinowicz et al. 2014). Rapid removal of sand reduces groundwater recharge and may result in premature failure of irrigation wells and associated problems in farming. It may also exert negative physical impacts on macro faunal communities. These include release of toxic materials either in association with actual mining or from machinery and materials used for mining (De Groot 1986; Newell et al. 2004). Water quality can be compromised by oil spills and leakages from excavation machinery and transportation vehicles. Such spills may poison aquatic life (Stebbins 2006). The main argument is that ‘Dredging causes an initial reduction in abundance, species diversity, and biomass of the benthic community in the extraction area’ (Trop 2017:78). Certain magnitudes of the extraction may result also in the lowering of the water table and subsequently water security issues (Pereira 2012).

Sand mining operations have also resulted in deforestation, habitat destruction and biodiversity erosion in some ecosystems (Saviour 2012). Large-scale sand mining operations have also seen damage to valuable timber resources and wildlife habitats (Stebbins 2006). Such impacts are said to be insurmountable if sand extraction activities are located on small river or stream than on a large stream (DID 2009). In addition to the size of the river, the shape of the river segment in which mining is done is also crucial in determining the magnitude of impact. Such observations hold that mining sand from a small straight channel with a narrow floodplain will have a much greater negative impact on the natural hydrologic processes than extracting sand on a braided channel with a wide floodplain (DID 2009).

Other studies have also observed that morphological changes in the exploited areas, noise, dust, and surface and groundwater pollution are the main environmental hazards associated with sand mining (Lambert 2001; Vintró et al. 2014). Another stream of research has focused on impacts associated with a specific type of sand mining. Kondolf and Swanson (1993), for instance, observed that ‘instream mining’ resulted in channel degradation and erosion, head cutting, increased turbidity, stream bank erosion and sedimentation of riffle areas. Associated adverse effects were noted on fish and other aquatic life. Stream sand mining has also been associated with adverse effects on stream geomorphology. Alterations in stream geomorphology resulted in infrastructure damage by undermining bridge piers and exposure of buried pipeline crossings and water supply intake (Kondolf 1997; Thrivikramaji 1986).

### Sand mining policy in South Africa

A regulatory system has been designed to govern all mining operations, including sand mining. Three main themes, namely mineral regulation, environmental regulation and land use planning regulation, can be discerned (Green 2012). Although these three are broader and much more generic to the otherwise wider and much more diverse mining industry, they are also applicable to the sand mining sector. Notable regulations include the *Mineral and Petroleum Resources Development Act 28 of 2000, National Water Act 36 of 1998, the National Environmental Management Act 1998* and a variety of land use planning regulations that govern the use of land as a resource in general. The current regulatory framework is not doing well in terms of serving what Green (2012) has identified as the three most important objectives, including conserving the resource; permitting an ordered and sustainable exploitation of the resource; and mitigating the environmental impacts associated with sand mining. Most regulatory instruments are either overlapping or in competition with each other. For example, the environmental concern mandate, as observed by Green (2012), seems to run across all regulatory instruments, making it difficult to tell which one should take precedence.We contend that an effective regulation can only be realised if there exists an explicit, well-established and strong regulatory system that is targeted specifically at sand mining operations. Such a regulatory framework should reflect specific policy intentions at three specific scales, including the strategic, institutional and operational levels.

## Methodology

### Study area

**FIGURE 1:**
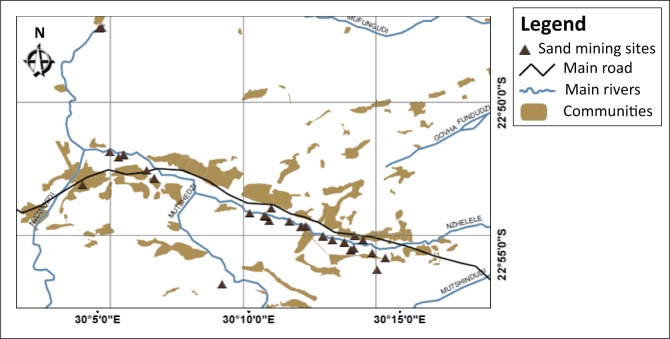
Spatial distributions of sampled sand mining sites.

We used specific sand mining sites along the Njelele River as our sampling unit of analysis to observe specific attributes that could be used to inform the several aspects of management policy that could be adopted to curb the negative physical impacts of sand mining. A total of 25 sites, as shown in Figure 1, were randomly selected.

Njelele River was chosen for analysis because of two main reasons. Firstly, it is a major watercourse in Limpopo Province of South Africa that supports a diversity of livelihood activities in many surrounding communities. Secondly, it possesses interesting attributes that, when analysed, would help inform the development of effective management policies. It is a catchment area spanning 2436 km^2^. The river meanders in a north-eastward direction across a wide plain rich in biodiversity, including numerous large mammals such as giraffes, white rhinos and blue wildebeests (Amponsah-Dacosta & Mathada 2017). In addition to sand mining, other dominant human activities around the river include cultivation (mostly on the floodplains) and livestock farming. In the recent past, communities along the river have been characterised by a surge in residential and commercial construction activities, which have seen a soaring demand for sand.

### Identification of areas of concern

To decipher major areas of concern that could inform the development of an effective regulatory and policy framework for sand mining, we borrowed insights from the commonly used approaches in the development of mining policies in general and specifically sand and gravel mining policies. For regulatory and policy-development-related matters, relevant studies have often employed three main methodologies, including empirical and Econometric Models (EEM), Material Flow Analysis (MFA) and System Dynamics (SD) (Arango-Aramburo et al. 2017).

The quasi-EEM model adopted in this study allowed us to use a combination of *K*-means clustering and discriminant function analysis (DFA) to determine morphological, ecological, socio-ecological, governance and physical attributes explaining the state of affairs in sand mining. Our focus on morphological impacts was not misplaced as other studies have indicated that morphological changes in the exploited areas are the main environmental hazards produced by small-scale mining operations such as sand mining (Lambert 2001; Vintró et al. 2014).

The MFA approach allowed us to distinguish between four processes that are normally involved when extracting natural resources in the mining sector, including (1) site clearance to remove vegetation, (2) resource extraction, (3) processing of the resource and (4) reclamation of the mined area (Schaetzl 1990). The SD component allowed us to examine the complex interaction between human and natural indicators. Our analysis did not seek to quantify such interactions because extensive research in that area already exists. We sought to draw important connections that existed at a more conceptual level. Such connections were then compared with our case study (i.e. the sand mining sites) through a qualitative methodology known as ‘pattern matching’.

### Pattern matching explained

Pattern matching was used to link two patterns, including the ‘theoretical pattern’ and the ‘observed pattern’. Three important steps, as proposed by Yin (2003), were employed.

***Step 1*:** Firstly, we specified a theory that we defined as a pattern of predicted events. Such a theory is composed by one or more hypothesis of the form ‘if X then Y’ held together by a set of rules or assumptions or both. To employ Kondolf, Smeltzer and Kimball (2001), characterisation of the relationship between quantity of sand extracted and the natural equilibrium of the channel, the resulting ‘if X then Y’ kind of a relationship may be expressed as a theoretical proposition:

IF there is excessive removal of sand (X) THEN the natural equilibrium of the stream will be distorted (Y). (n.p.)

Similarly if we were to employ characterisation of the relationship between sand extraction method and its effect on macro faunal communities (Bagchi 2010), the resulting ‘if X then Y’ kind of a relationship may be expressed as a theoretical proposition:

IF machines are extensively used to extract sand (X) THEN serious negative consequences on macro faunal communities will result (Y). (n.p.)

Table 1 lists the details on study variables and research assumptions that governed a series of such ‘if X then Y’ kind of relationships.

**TABLE 1:** Study variables and associated assumptions

Layer	Variable	Research assumption(s)	Supporting references
Morphological	Quantity of sand extracted	- Certain magnitudes of the extraction may result in the lowering of the water table and subsequently water security issues. - Excessive removal of sand may distort the natural equilibrium of a stream channel. - Excessive removal of sediment than what system can replenish will result in negative environmental impacts. - If sand is extracted rapidly, groundwater evaporates fast, reducing groundwater recharge, increasing initial and premature failure of irrigation wells and the associated problems in farming.	Collins & Dunne 1990; Goddard 2007; Kondolf et al. 2001; Langer 2003; Pereira 2012.
	Observed depth changes	- Bed degradation can undermine bridge supports, pipe lines or other structures. - Extraction of bed material in excess of replenishment by transport from upstream causes the bed to lower (degrade) upstream and downstream of the site of removal - Degradation can deplete the entire depth of gravelly bed material, exposing other substrates that may underlie the gravel, which could in turn affect the quality of aquatic habitat. - Rapid bed degradation may induce bank collapse and erosion by increasing the heights of banks.	Collins & Dunne 1990; Thrivikramaji 1986.
	Size and shape of channel segment	- Mining sand from a small straight channel with a narrow floodplain will in principle have a greater impact on the natural hydrologic processes than mining sand in a braided channel with a wide floodplain.	DID 2009; Kelly, Ramsey & Byrnes 2004
Physical/engineering	Extraction method	- Some sand mining extraction methods such as the use of machinery have serious negative consequences on macro faunal communities. - Mechanical methods of sand extraction may release toxic materials either in association with actual mining or from incidental or accidental releases from machinery and materials used for mining. - Use of shovels (which have less impact on the ground) is recommended over use of heavy front end loader equipment (which may have serious environmental consequences).	Bagchi 2010; De Groot 1986; Kondolf 2007; Kondolf et al. 1997; Mwangi 2007; Newell et al. 2004; Stebbins 2006; Troy 2017
	Type of sand-mining activity	- Instream sand mining has adverse effects on aquatic life through direct harm inflicted on the organisms or through habited degradation. - Instream sand mining can also disrupt the food-web that is so critical in supporting aquatic life. - Alterations in stream geomorphology as a result of instream sand mining can result in damage to critical infrastructure. - Instream sand mining causes destruction of aquatic and riparian habitat through large changes in channel morphology, lowering of water table, instability and sedimentation at mining sites.	Bagchi 2010; Kondolf 2007; Mwangi 2007; Newell et al. 2004; Stebbins 2006.
	Distance from mining site	- Villagers located near mining sites are likely to suffer most from noise and air pollution. - Air pollution caused by dust particles can be a health hazard to nearby communities as it may result in respiratory disorders such as asthma and irritation of lungs.	Lawal 2011; Saviour 2012; Wokorach 2002.
	Distance from regular water source	- Ecological and human health consequences of sand mining are likely to be greater where extraction takes place near domestic water sources.	Lawal 2011; Saviour 2012; Wokorach 2002.
	Location of extracting site in relation to floodplain	- Sand mining sites located outside active floodplain cause minimum environmental damage. - Extraction sites located on one side of floodplain minimises the compaction of active channels with heavy tipper trucks and front end loaders. - Locational behaviours that permit crossing of active channels with trucks may lead to contamination of water with oil spills and leakages.	Hill & Kleynhans 1999; Lawal 2011; Schaetzl 1990.
Ecological	Area of disturbance	- Sand mining operations result in deforestation, habitat destruction and biodiversity erosion. - Continuous sand mining may result in complete removal of vegetation and destruction of topsoil and subsoil, and subsequently a reduction in faunal population.	Pereira 2012; Saviour 2012; Stebbins 2006.
Socio-environmental	Adoption of environmentally responsible practices	- Adoption of environmentally responsible practices by sand miners will lead to sustainable outcomes. - Making issues of environmentally responsible practices a compliance matter may help the industry actors to minimise the negative impacts of their operations on the environment and may also improve their accountability with regard to environmental issues.	Driussi & Jansz 2006; Kapelus 2002; Troy 2017; Vintró et al. 2014; Worrall et al. 2009.
	Adoption of pro-active environmental practices	- Proactive environmental practices in the sand mining industry will permit sustainable competitive improvements.	Barba-Sánchez & Atienza-Sahuquillo 2010.
	Observable social benefits	- Sand management may be improved by adopting extraction policies aimed at reducing negative impacts and increasing social benefits.	Troy 2017
	Environmental training	- Environmental training may assist some sand mining establishments to improve their accountability in relation to environmental issues.	Driussi & Jansz 2006.
	Adoption of pollution prevention mechanisms	- Adoption of pollution prevention technologies may assist some sand mining establishments to improve their accountability in relation to environmental issues.	Driussi & Jansz 2006.
	Adoption of green business practices	- Adoption of green business practices may assist some sand mining establishments to improve their accountability in relation to environmental issues.	Driussi & Jansz 2006; Fernández & Melé 2005; Hilson & Nayee 2002.
Socio-economic	Employment generation	- Licensed sand mining activities can create employment opportunities.	Mbaiwa 2008; Lupande 2012.
	Wealth creation	- Sand and gravel activities generate revenue and income to local governments and land owners through payments of licenses.	Mwangi 2007; Lawal 2011; Saviour 2012.
Governance	Accountability to environmental concerns	- Existence of an accountability framework will compel sand miners to take environmentally responsible actions. - Sand mining industry actors are more willing to adopt environmentally friendly practices if such actions are matters of compliance with existing laws or regulations	Claver et al. 2004; Driussi & Jansz 2006; Kapelus 2002; Troy 2017; Vintró et al. 2014; Wheeler et al. 2002.
	Existence of comprehensive environmental management systems	- Existence of ‘comprehensive environmental management systems’ may ensure that the welfare and well-being of the current generation is promoted without compromising the quality of life of future generations. - Sand mining companies that implement comprehensive environmental management systems are able to effectively anticipate environmental problems and to secure the much needed support of both concerned national governments and local communities. - Sand management may be improved by adopting extraction policies aimed at reducing negative impacts	Azapagic 2004; Hilson & Nayee 2002; Vintró et al. 2014
	Adoption of ethical and sustainable policies	- Adoption of specific codes of conduct may assist some sand mining establishments to improve their accountability in relation to environmental issues.	Azapagic 2004; Vintró et al. 2014
	Codes of conduct dedicated to sand mining operations	- Adoption of specific codes of conduct may assist some sand mining establishments to improve their accountability in relation to environmental issues.	Driussi & Jansz 2006; Fernández & Melé 2005; Hilson & Nayee 2002.

***Step 2*:** The events in the theory pattern then acted as a series of benchmarks against which actual data from the case study (sand mining sites) were compared. Information on all case study events was collected through observation, a series of transect walks, interaction with communities and a sample of sand miners. A household survey was conducted with 100 households residing near the sampled sites. Actual measurements on a number of morphological, physical and ecological variables associated with each sand mine were taken and recorded. Such empirical evidence generated was collected on the basis of the indicator system developed (Table 1), which provided a useful basis for building policy guidelines targeted at regulating the sand mining industry – an attribute that has been acknowledged by some studies in the mining sector in general (Haberl et al. 2004).

Interactions with selected community members and sand minors were guided by a series of participatory focus-group discussions. Such a participatory way of gathering information was found to be important because developing an effective regulatory environment arguably requires an in-depth understanding of the interaction and interdependence between technical issues, the environment, stakeholders and policy-makers in the mining sector (Arango-Aramburo et al. 2017). The basic concept of Participatory Rural Appraisal (PRA) approach, as espoused by Chambers (1992), was therefore adopted. Data from PRA studies were consolidated using important PRA tools such as the influence and importance matrix (IIM), participatory time series analysis, and matrix scoring and ranking. Views generated at the participatory stage were combined with actual technical measurements of the impact of sand mining activities and data from MFA.

**FIGURE 2:**
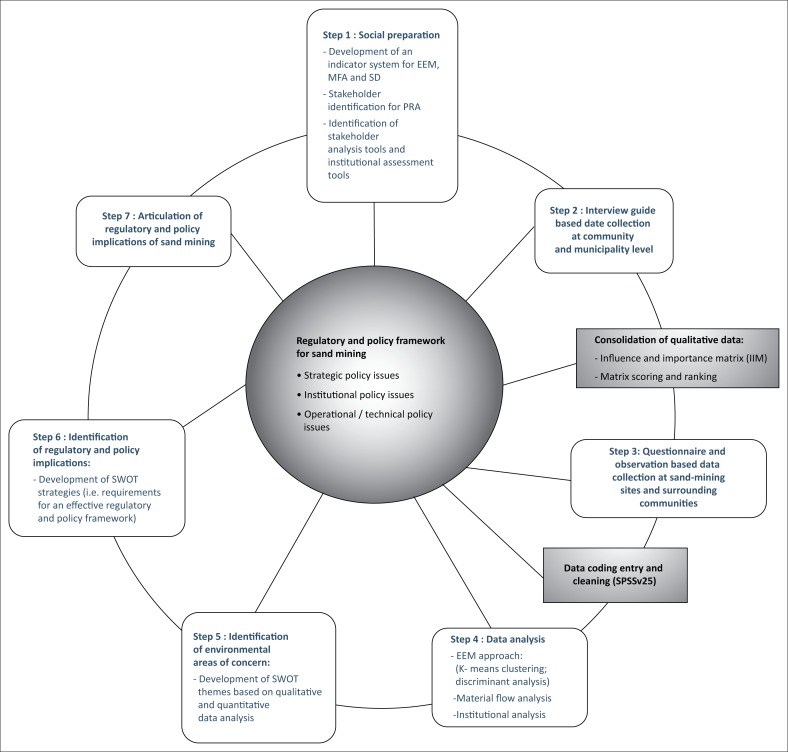
Adopted research plan.

***Step 3***: As a final step, both patterns were matched by analysing whether the events in the case study pattern were in line with the events in the theory pattern. A combination of Gap analysis and Strengths, Weaknesses, Opportunities and Threats (SWOT) analysis were used to identify main environmental areas of concern and to subsequently articulate regulatory and policy implications of sand mining at three levels, including strategic, institutional and operational levels. The use of SWOT analysis in mining studies is not new. Nikolaou and Evangelinos (2010), for instance, conducted a SWOT analysis of the Greek mining industry and results were used to develop sustainable mining practices. Figure 2 summarises the overall methodology that enabled us to comprehend sand mining as a system, including the interactions among its different elements.

## Ethical consideration

This article followed all ethical standards for a research without direct contact with human or animal subjects.

## Results and discussion

**FIGURE 3:**
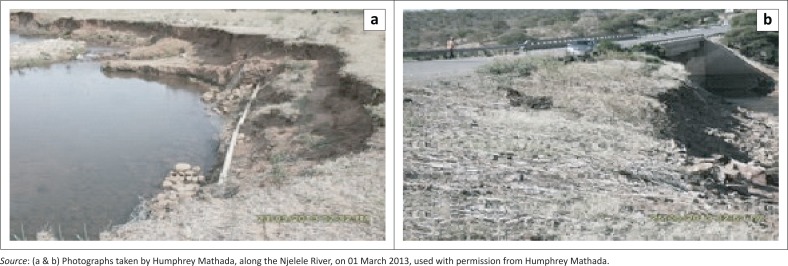
Exposed water line (a) and bridge foundation (b).

Our analysis found that the main type of activity practised along the Njelele River is instream mining. Seventy-two per cent of the sampled sites were under instream mining, whereas 28% were under floodplain mining. Such statistical values are disturbing because most studies have revealed that instream mining causes serious morphological and ecological disturbances if not conducted under a robust regulatory framework (Bagchi 2010; De Groot 1986; Kondolf 2007; Mwangi 2007; Newell et al. 2004; Stebbins 2006). We also observed that alterations in stream geomorphology occurred as a result of instream sand mining. Such alterations resulted in serious damage to critical infrastructure, such as water lines and bridges (Figure 3).

**FIGURE 4:**
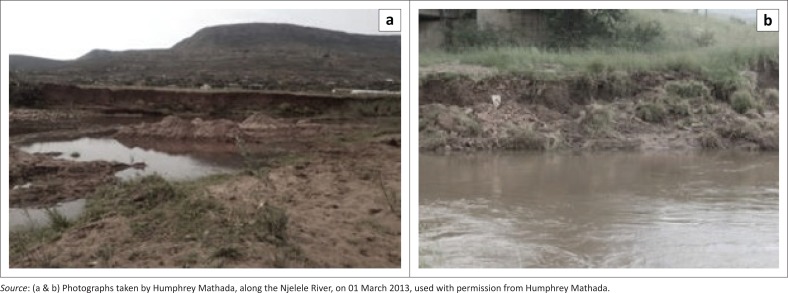
Evidence of (a) erosion at selected (b) reaches along the Njelele River.

Study results also show that the main method of sand extraction practised by sand miners is manual. About 72% rely on manual means of extraction, whereas 28% of sand mining operations rely on mechanical means. The use of mechanical extraction is worrisome because a significant number of studies have revealed a host of negative environmental consequences associated with this method (Kondolf 2007; Mwangi 2007; Newell et al. 2004; Stebbins 2006). We observed instances where manual mining of sand located on active flood plain resulted in complete removal of vegetation and destruction of topsoil and subsoil, subsequently causing bank erosion (Figure 4). Removal of topsoil has greatly compromised the scenic landscape and the permanent loss of soil and habitat.

In order to garner an in-depth and consolidated understanding of challenges associated with each cluster of sand mining sites, we performed a *K-*means clustering using a sample of critical morphological, ecological and socio-ecological variables. Because data were captured using different scales, we first standardised the data set.

**FIGURE 5:**
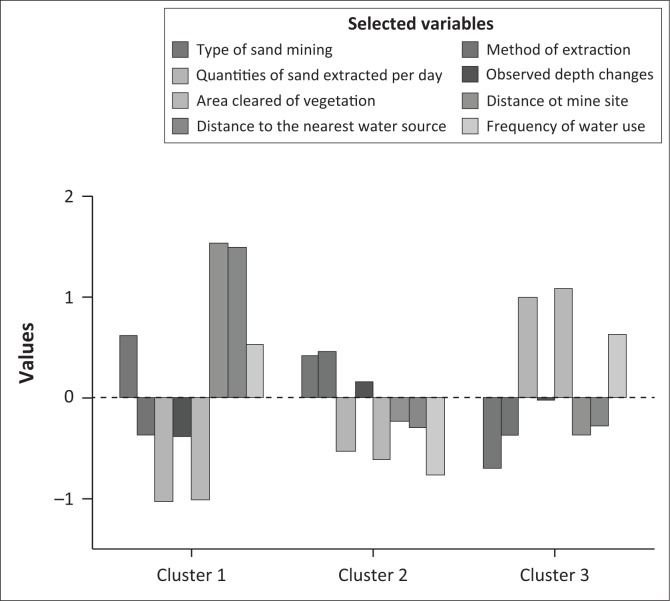
Sand mining clusters along the Njelele River.

Results from *K*-means clustering showed three distinct clusters (Figure 5). Cluster one comprised four sand mining sites, cluster two had 11 and cluster three had 10. By implications, such three distinct clusters are expected to display different environmental concerns that would therefore not require the ‘one-size-fits-all’ kind of solutions.

**FIGURE 6:**
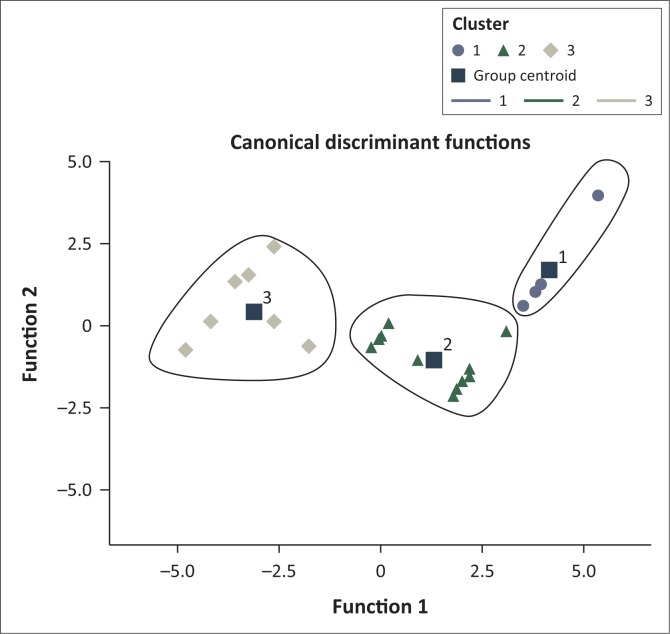
Canonical discriminant function graph.

We carried out a DFA to explore both the relative stability of the cluster solution arrived at and significant cluster-defining attributes. As shown in Figure 6, DA revealed that all sand mining sites belonged to three clusters. No overlaps were envisaged as all points representing sand mining sites were clustered around a unique group centroid.

The analysis showed that 10 sand mining sites belonging to cluster 3 are severely constrained in terms of morphological, ecological and socio-ecological impacts. The second-most constrained cluster is cluster two (comprising 11 sites). Sand mining sites in cluster 3 are characterised by high volumes of sand extracted at any given day. Despite the main method of sand extraction being manual, we found that the frequency of extraction was high because such activities are located near the surrounding communities where demand for this resource is very high. Approximately 11 900 m^3^ of sand is extracted in such sites per month. Such excessive extraction activities have compromised greatly the scenic landscape. Similar to observations shared by Goddard (2007), they have in most cases caused hillside erosion, accumulation of water upstream, permanent loss of soil as well as the destruction of natural habitat.

Data from the field revealed that most of the extractions sites in both cluster 2 and cluster 3 are located either on the active river channel or within 500 m from the main river channel and on riparian area disturbing the entire river ecosystem functionality. Severe morphological and ecological stream disturbances in cluster 3 are associated with dominant instream sand mining activities. Such activities have resulted in serious depth changes when compared with other clusters. The amount of vegetation cleared as a result of sand mining activities was also found to be the highest followed by that of cluster two sand mining sites.

**FIGURE 7:**
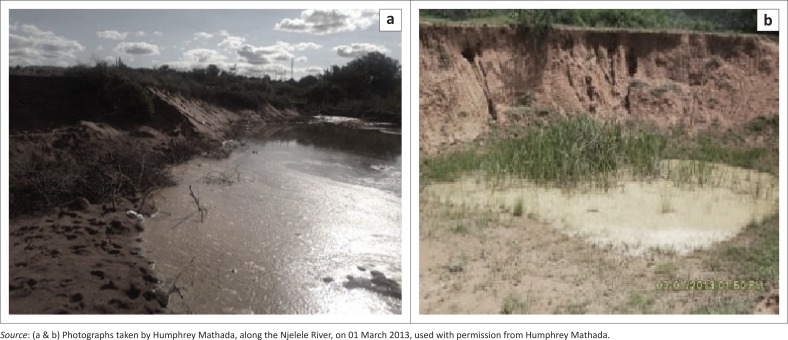
(a & b) Turbidity issues in selected sand mining sites.

Because of the relatively short distance between sand mining sites and areas where communities fetch water for domestic use and for recreational activities such as swimming, we found sand mining activities in this cluster to be posing serious health challenges to the communities, as water frequently sourced is usually contaminated (Figure 7).

We also observed that a number of attributes have caused cluster one sand mining sites to have the least possible morphological and ecological impacts. All four sites belonging to this cluster were characterised by flood plain sand extraction. Floodplain extraction in three of the four sites was set back from the main channel. This in principle is a welcome development as empirical studies elsewhere have shown that less environmental impacts are associated with sand mining activities located outside active floodplain (Lawal 2011; Schaetzl 1990). Unlike in some of the cluster two sites, we did not find location-related activities that permitted crossing of active channels with trucks and possibly leading to contamination of water with oil spills and leakages (Lawal 2011). In all four recorded cases, the maximum depth of excavations in cluster one sites has remained above the channel thalweg, as recommended by most studies (Kondolf 2007; Newell et al. 2004; Stebbins 2006) – an attribute that has been violated in a considerable number of extraction activities associated with both cluster two and cluster three sites. We also observed several other factors that made cluster one sites to have the least possible impact. In two cases, stockpiles of sand extracted were located at a distance further away from the active river channel – an attribute that has been violated mostly in cluster two and cluster 3 sites. All of the four extraction sites observed were taking place on a smaller segment of the river channel – a characteristic having minimum impact on the environment and river ecology, as touted in several studies (DID 2009). An almost similar trend was observed on five sites of cluster two, where extraction activities were located on relatively straight channels in narrow floodplains. Morphological and ecological impacts under such circumstances are expected to be lower than in cluster three sites, where most sand mining activities are located on a braided channel with a wide floodplain where degradation levels are high owing to increased erosion activities (Goddard 2007; Kondolf 2007).

Data from DA and *K*-means clustering and ANOVA test results (Table 2) revealed several variables that were significantly shaping some of the morphological, ecological and socio-ecological outcomes associated with specific sand mining clusters.

**TABLE 2:** ANOVA test results associated with *K*-means clustering.

Variables	Cluster		Error		*F*	Sig.
	Mean square	*df*	Mean square	*df*		
Type of sand mining	4.121	2	0.716	22	5.754	0.010
Method of extraction	2.083	2	0.902	22	2.310	0.123
Quantities of sand extracted per day	8.629	2	0.306	22	28.160	0.000
Observed depth changes	0.438	2	1.051	22	0.417	0.664
Area cleared of vegetation	9.916	2	0.189	22	52.341	0.000
Distance from communities to mine site	5.628	2	0.579	22	9.716	0.001
Distance from the nearest water source	5.332	2	0.606	22	8.795	0.002
Frequency of water use	5.781	2	0.565	22	10.226	0.001

**FIGURE 8:**
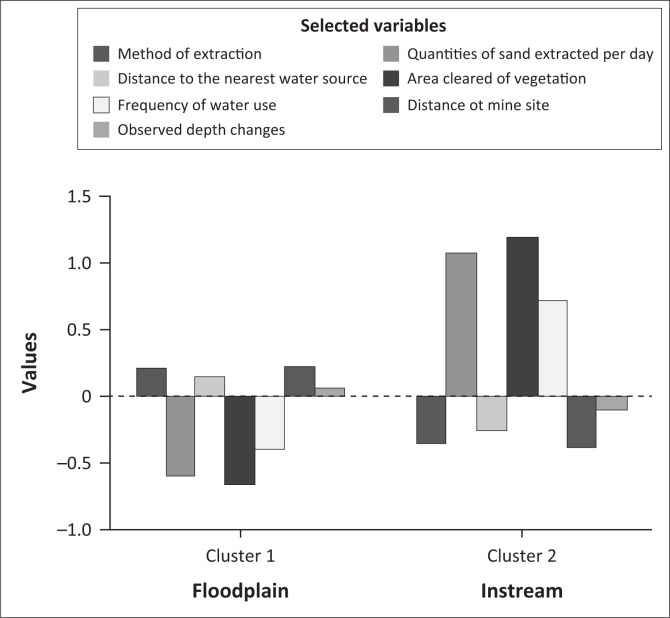
*K*-means clustering by type of sand mining activity.

Critical variables with *p*-values less than 0.05 included the type of sand mining activity, quantities of sand extracted, area cleared of vegetation, distance from nearby communities to the mining site, distance from mining site to the nearest community water source and frequency of water taken from the river, by communities. We posit that management interventions should pay special attention to these variables. We also posit that policy interventions should be specified for each type of sand mining activity. This is because the morphological, ecological and socio-ecological attributes were found to significantly vary according to the type of sand mining activity (i.e. instream or floodplain). Results from *K*-means clustering performed on standardised values of selected variables showed significant differences between instream and floodplain sand mining (Figure 8). Impact bearing variables such as volume of sand extracted per day and area cleared of vegetation are clearly high for instream as opposed to flood plain sand mining.

ANOVA test results revealed that the most critical distinguishing variables (*p* < 0.005) were quantities of sand extracted per day, area cleared of vegetation and how frequent households would access water resources near sand mining sites.

It was however possible to decipher some common attributes in terms of the perceived social impacts of both types of sand mining. A participatory historical time series analysis showed several challenges posed by sand mining activities (Table 3).

**TABLE 3:** Participatory historical time series analysis of sand mining impacts.

Variable of interest	Community perceptions over time†		
	In the last 20 years	In the last 10 years	In the last 5 years
Sand mining activities	- There were few sand mining activities operating along the river	- We witnessed a steady increase in the number of sand-mining activities	- We witnessed the biggest surge in sand mining activities
Water quality	- We used to fetch clean water for domestic consumption	- Water for domestic consumption started changing colour.	- The rate of deterioration in water quality increased sharply in recent times
River width and depth	- The rivers was so big that it was difficult to cross on foot	- The river gradually shrunk to the extent that villages could walk across in some sections	- The river has further shrunk to the extent that almost anyone can cross it at any point.
Vegetation cover	- there was dense vegetation cover near the river	- Vegetation started to disappear fast as a result of increasing sand mining operations	- There is virtually no vegetation around areas where sand mining operations are located.
Fishing activities	- We used to catch fish with a high degree of ease because of their abundance	- Catching fish became a bit challenging as water levels receded because of sand mining activities	- We are now forced to walk great distances to catch fish in areas far away from sand mining activities.
Swimming activities	- There were so many places along the river that community members could go and swim.	- The number of places where communities could go and swim started dwindling	- It is now difficult to find a safe and clean place to go and swim. We are now forced to travel very long distances in search for clean and safe places to swim.

† , Based on perceptions shared by a sample of community members during focused group discussion sessions.

**FIGURE 9:**
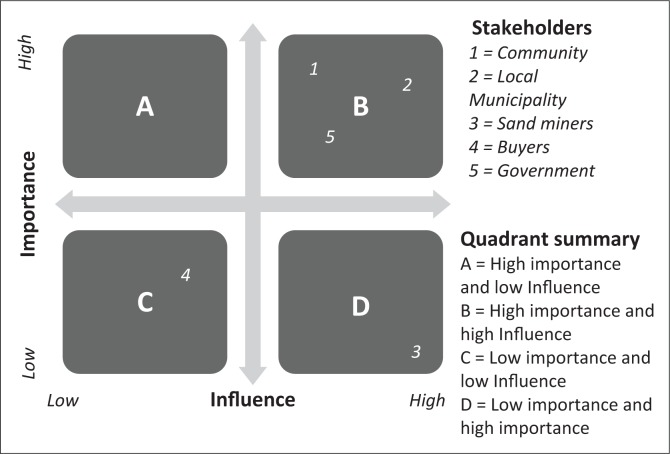
Influence and importance matrix.

Results in Table 3 reveal that the major impacts brought about by a sharp increase in the sand mining activities over the last 20 years have been deterioration in water quality, morphological changes in the river channel, destruction of vegetation, a reduction in fishing activities as well as recreational activities such as swimming. In an attempt to find out what should be done by whom to overcome a series of social challenges brought by sand mining, communities were asked to identify and rank key stakeholders according to their importance and influence at the focus-group discussion stage. Results are summarised in Figure 9.

In order to reverse the negative environmental consequences associated with sand mining, community members recommended that important stakeholders (including local community structures, the government and the local municipality) should collaborate and use their high influence to control and manage the activities of sand miners that were deemed to cause harm to the environment.

### Regulatory and policy implications

Decisions concerning where to mine as well as how much and how often to extract need to be determined by policy or specific guidelines. We recommend development of specific policies or guidelines targeted at influencing several variables associated with sand mining at least at the operational level. To do this, we borrow insights from problems associated with sand mining that have been discussed in the preceding section. Through Gap analysis and the development of a SWOT matrix, we were able obtain data through PRA and quantitative analysis by generating a series of challenges and opportunities associated with sand mining activities along the Njelele River. Such challenges and opportunities were developed according to the three cluster solution generated using the *K*-means clustering approach (Box 1). A Threats, Opportunities, Weaknesses and Strengths (TOWS) strategy matrix upon which the discussion of regulatory and policy implications is built was developed on the basis of the identified challenges and opportunities.

BOX 1 Strengths, weaknesses, opportunities and threats.

**Table:** 

Strengths (S)	Applicable to cluster descriptor				Weakness (W)	Applicable to cluster descriptor			
	N/A	1	2	3		N/A	1	2	3
1. Some minors are aware of the need to protect the environment.		√	√		1. Over-reliance on mechanical means of sand extraction				√
2. Over-reliance on manual means of sand extraction		√√	√√	√	2. Excessive levels of sand extraction.		√√	√	√
3. Floodplain extraction in a notable number of sites set back from the main channel.		√	√		3 Floodplain extraction in a notable number of sites is not set back from the main channel		√	√	√
4. Sand mining sites located outside active floodplain		√√	√		4 Sand mining sites located on active floodplain		√√	√	
**Opportunities (O)**	Applicable to cluster descriptor*				**Threats (T)**	**Applicable to cluster descriptor**			
	**N/A**	**1**	**2**	**3**		**N/A**	**1**	**2**	**3**
1. Share of income taken home for own consumption (by community) owing to their involvement in sand-mining activities.					1. Lack of motivation/interest/self-drive by sand miners to protect the environment.		√√	√√	√√
2. Economic benefits through sale of sand.		√	√	√	2. Absence of explicit short-term and/or long-term monitoring program to monitor sand mining activities.		√√	√√	√√
3. Skills and knowledge spill-over to the community gained through working in sand mining activities.		√√	√√	√√	3. Absence of an annual status and/or trends report.	√√	√√	√√	√√
4. Potential revenue source for the municipalities through licensing of mining activities.	√√	√	√	√	4. Knowledge of minimal acceptable physical and biological condition of a channel for major rivers is missing at both district and national levels.		√√	√√	√√

NB: Only a sample of variables is presented. In total, 13 strengths, 20 weaknesses, 18 opportunities and 11 threats were identified.

* , Cluster descriptor number informed by *K*-means cluster solution presented earlier on in the analysis.

N/A: Not applicable to any of the sand mining sites in the cluster.

√ , Applicable to some sand mining sites in the cluster.

√√ , Applicable to all sand mining sites in the cluster.

One of the recommendations we are putting forward is the development of a policy that explicates what Goddard (2007) has referred to as a reference state. A reference state is the minimal acceptable physical and biological condition of a channel (Goddard 2007). As observed by Goddard (2007), its determination should follow an in-depth study that seeks to explore fluvial processes associated with the river at various sand mining sites. Associated with the reference state and a more strategic level is the need to come up with a sand budget. Such a sand budget should be developed for a specific extraction site in order to determine the amount of sand that can be removed without causing environmental degradation and other associated consequences such as bank failure. Because sand extraction methods have important repercussions for the degree of degradation, we recommend that the sand budget take cognisance of extraction methods because mechanical methods of sand extraction were found to be the major cause of excessive extraction behaviours particularly for cluster two and cluster three sand mining sites.

To complement such a sand budget, we recommend implementation of measures that will help detect and deter excessive sand extraction behaviours. This can be done at a more strategic level by implementing continuous monitoring of sand mining activities. We envisage that the environmental integrity of the river ecosystem will be enhanced further if at a more strategic level government take lead in the development of a monitoring system as part of a comprehensive environmental management plan. Such comprehensive environmental management plans should be implemented by sand miners as a matter of compliance.

We also propose developing a comprehensive environmental management system that controls all sand mining operations. The existence of ‘comprehensive environmental management systems’ may ensure that the welfare and well-being of the current generation is promoted without compromising the quality of life of future generations (Azapagic 2004; Vintró et al. 2014). This is because studies elsewhere have shown that sand mining companies that implement comprehensive environmental management systems are able to effectively anticipate environmental problems and to secure the much needed support of both concerned national governments and local communities (Azapagic 2004; Hilson & Nayee 2002; Vintró et al. 2014). The implementation of such systems should be a matter of compliance if they are to bear results. This is because empirical evidence from elsewhere has shown that sand mining industry actors are more willing to adopt environmentally friendly practices if such actions are matters of compliance with existing laws or regulations (Driussi & Jansz 2006; Kapelus 2002; Troy 2017; Vintró et al. 2014). Absence of such laws that are dedicated to the sand mining industry has however seen most minors not complying with the existing generic mining-industry-wide legislation. Such non-compliance has been high among unlicensed sand minors. At an institutional level, government through municipal bylaws need to strengthen development control measures by making sure that unlicensed minors are held accountable for their actions. The licensing system needs to be further improved so that all mining activities are licensed. Additional revenue generated through licensing can further be used to protect the environment through investments in environmental awareness campaign programmes.

In addition to the environmental management system, there is a need to develop a sand-mining-industry-specific code of conduct that should punish bad behaviour and reward good practice. Such a code of conduct should be complemented by the introduction of accountability frameworks that will allow sand minors to adopt environmentally responsible practices (DID 2009).

We also observed (particularly for cluster three sites) that excessive extraction behaviours were also associated with sites located close to the communities with the shortest average distance of 380 m. These results do not come as a surprise, as Stebbins (2006) observed a similar behaviour in the United States. Such a practice of locating sand mining sites close to consumer as Stebbins (2006) observed is justified because sand is a bulky commodity that cannot economically stand costs of long-distance transportation. We therefore recommend the development of guidelines that require extraction activities to be located further away from the communities.

Taking advantage of income generated through involvement in or licensing of sand mining activities, we recommend the introduction of community-wide environmental training programmes. Such programmes should help sensitise sand miners on benefits associated with protecting the environment. Environmental awareness and training may also help in calculating a culture of environmental consciousness by sand minors and the surrounding communities at large. The need to adopt sustainable practices in the sand mining business should be the main emphasis in such training and environmental awareness programmes. We envisage that training programmes that promote sustainable mining behaviours will help avoid bad mining practices such as excessive sand extraction behaviours, not setting back flood extraction from main channel, locating sand mining sites on active floodplain, not setting the maximum depth of floodplain extraction below channel thalweg, permitting crossing of active channel by heavy equipment, locating extraction sites near critical infrastructure, not aligning sand extraction to known replenishment rate of the river and locating sand mining activities on braided channel with a wide floodplain and at times on large segment of the river channel among others.

Environmental sustainability programmes alone are not sufficient. A number of complementary policy guidelines need to be developed. Environmental consequences of floodplain extraction, for example, can be reduced by developing guidelines that require such sand mining activities to be set back from the main channel. There is also an urgent need to develop guidelines that require the maximum depth of floodplain extraction to be maintained above the channel thalweg. This practice alone may help limit environmental degradation and morphological consequences that are associated mostly with floodplain extraction. Discouraging crossing of active channels by heavy equipment may limit compaction of the river bed. This can only be made possible by developing guidelines that would restrict sand extraction activities to only one side of the active channel. The problem of soil and riverbed erosion can be minimised by developing policy guidelines that require the side slopes of floodplain excavation to be located within an acceptable range. Sand management may also be improved by adopting extraction policies aimed at reducing negative impacts. This can be done, for example, by discouraging mechanical means of sand extraction in certain sections of the river system where the associated river ecosystem is deemed to be very fragile. Manual means of sand extraction may instead be promoted subject to continuous monitoring targeted at matching extraction rate with the replenishment capacity of the river system.

In the long term, we recommend that alternative inputs to the construction industry should be explored so that demand for sand is reduced. For instance, Kuttipuran (2006) has noted that reliance on concrete structures in the construction industry can be lowered if households are encouraged to use wood as an alternative resource. Other long-term measures would include exploring other employment generation methods so as to limit the involvement of communities in sand mining activities.

## Conclusion

The main mandate of this analysis was to discuss regulatory and policy implications of sand mining activities along the Njelele River in the Limpopo Province of South Africa. This was done against the background that existing mineral, environmental and land use planning regulations are broader and much more generic to the otherwise wider and much more diverse mining industry and therefore have been less effective in controlling operations in the sand mining sector. More specifically they have not been effective in conserving sand as a resource; permitting an ordered and sustainable exploitation of the resource; and mitigating the environmental impacts associated with its mining. This analysis argued that an effective regulation can only be realised if there exists an explicit, well-established and strong regulatory system that is targeted specifically at sand mining operations. The analysis has been inspired partly because of the need to address concerns relating to where to mine, how much to extract, and how often the extraction process should take place and partly because South Africa lacks specific guidelines targeted at controlling the activities of sand minors particularly at the operational level. Several policy or regulatory recommendations have been put forward on the basis of morphological, physical, ecological and socio-ecological circumstances surrounding a sample of such operations along the Njelele River.
