# Corrigendum: Regulatory and policy implications of sand mining along shallow waters of Nzhelele River in South Africa

**DOI:** 10.4102/jamba.v11i3.901

**Published:** 2019-12-06

**Authors:** Tendayi Gondo, Humphrey Mathada, Francis Amponsah-Dacosta

**Affiliations:** 1 Department of Urban and Regional Planning, School of Environmental Sciences, University of Venda, Thohoyandou, South Africa; 2 Department of Geography and Geo-Information Sciences, School of Environmental Sciences, University of Venda, Thohoyandou, South Africa; 3 Department of Mining and Environmental Geology, School of Environmental Sciences, University of Venda, Thohoyandou, South Africa

In the version of this article published earlier, the name of the river Nzhelele was incorrectly spelt as Njelele. The river name has been corrected in the article title and in the ‘How to cite this article’ section. The authors apologise for any inconvenience caused.

